# Euthanasia and assisted death from a Spanish perspective

**DOI:** 10.1192/j.eurpsy.2025.194

**Published:** 2025-08-26

**Authors:** M. Martin-Carrasco

**Affiliations:** Psychiatry, Clinica Psiquiatrica Padre Menni, Pamplona, Spain

## Abstract

**Abstract:**

Medical assistance in dying is an increasingly available option for people suffering (solely) from psychiatric disorders. Initially promoted to alleviate the suffering of terminally ill people, a growing number of jurisdictions are adopting it for any cause of intractable and severe suffering, including mental disorders. Today, the BENELUX countries, along with Spain and Switzerland, explicitly authorise it or allow it de facto. Other countries, such as Canada, are considering implementing it. Although in jurisdictions where it is permitted it is argued that it is discriminatory not to consider mental suffering as sufficient cause, there are reasons for concern. The procedure is likely to be used as an alternative to care, that is, as a gentler form of suicide, more commonly used by women. The long-term impact of this practice must be considered, as it sends the message that mental illnesses may not be curable, and that it is not worth the effort to treat them, or to demand the necessary care. Furthermore, all of these factors must be considered in the context of highly stigmatized disorders to which clearly scarce resources are allocated.

**Disclosure of Interest:**

None Declared

